# Indirect determination of biochemistry reference intervals using outpatient data

**DOI:** 10.1371/journal.pone.0268522

**Published:** 2022-05-19

**Authors:** Luisa Martinez-Sanchez, Christa M. Cobbaert, Raymond Noordam, Nannette Brouwer, Albert Blanco-Grau, Yolanda Villena-Ortiz, Marc Thelen, Roser Ferrer-Costa, Ernesto Casis, Francisco Rodríguez-Frias, Wendy P. J. den Elzen

**Affiliations:** 1 Clinical Laboratories, Biochemistry Department, Vall d’Hebron University Hospital, Barcelona, Spain; 2 Department of Clinical Chemistry and Laboratory Medicine, Leiden University Medical Centre, Leiden, The Netherlands; 3 Departament de Bioquímica i Biologia Molecular, Universitat Autònoma de Barcelona, Bellaterra, Spain; 4 Department of Internal Medicine, Section of Gerontology and Geriatrics, Leiden University Medical Center, Leiden, The Netherlands; 5 Diagnost-IQ, Expert Centre for Clinical Chemistry, Purmerend, The Netherlands; 6 Laboratory for Clinical Chemistry and Hematology, Amphia, Breda, The Netherlands; 7 Stichting Kwaliteitsbewaking Medische Laboratoriumdiagnostiek, Nijmegen, The Netherlands; 8 Department of Laboratory Medicine, Radboud University Medical Centre, Nijmegen, The Netherlands; 9 Atalmedial Diagnostics Centre, Amsterdam, The Netherlands; 10 Department of Clinical Chemistry, Amsterdam Public Health research institute, Amsterdam UMC, Amsterdam, The Netherlands; Inselspital, Bern University Hospital, SWITZERLAND

## Abstract

The aim of this study was to determine reference intervals in an outpatient population from Vall d’Hebron laboratory using an indirect approach previously described in a Dutch population (NUMBER project). We used anonymized test results from individuals visiting general practitioners and analysed during 2018. Analytical quality was assured by EQA performance, daily average monitoring and by assessing longitudinal accuracy between 2018 and 2020 (using trueness verifiers from Dutch EQA). Per test, outliers by biochemically related tests were excluded, data were transformed to a normal distribution (if necessary) and means and standard deviations were calculated, stratified by age and sex. In addition, the reference limit estimator method was also used to calculate reference intervals using the same dataset. Finally, for standardized tests reference intervals obtained were compared with the published NUMBER results. Reference intervals were calculated using data from 509,408 clinical requests. For biochemical tests following a normal distribution, similar reference intervals were found between Vall d’Hebron and the Dutch study. For creatinine and urea, reference intervals increased with age in both populations. The upper limits of Gamma-glutamyl transferase were markedly higher in the Dutch study compared to Vall d’Hebron results. Creatine kinase and uric acid reference intervals were higher in both populations compared to conventional reference intervals. Medical test results following a normal distribution showed comparable and consistent reference intervals between studies. Therefore a simple indirect method is a feasible and cost-efficient approach for calculating reference intervals. Yet, for generating standardized calculated reference intervals that are traceable to higher order materials and methods, efforts should also focus on test standardization and bias assessment using commutable trueness verifiers.

## Introduction

Specialists in clinical chemistry should provide accurate and useful information into their clinical laboratory reports. Reference intervals are commonly presented together with the actual analytical results. Their correct evaluation is crucial due to their use as a clinical decision-making tool [1]. Most manufacturers provide reference intervals in their technical documentation. According to ISO15189:2012, it is the responsibility of the laboratory to either validate them, find reference intervals from other sources or calculate the appropriate reference intervals for their method and population. Two different approaches to calculate reference intervals could be used: (a) The procedure recommended by the International Federation of Clinical Chemistry (IFCC), known as the direct method and [2,3] (b) an alternative approach, known as the indirect method [4].

The direct approach uses a bottom-up strategy. In this sense, the reference population will be analysed in detail in order to unravel their characteristics and then a realistic “model” will be constructed to derive the distribution of the reference population and the reference intervals. This methodology has been widely used and standardized [2], but it is laborious and expensive. In addition, it struggles with selection bias, in combination with subjective terms as “reference population” and “health” [5]. As an alternative approach, the indirect method uses a top-down approach. It starts by acquiring a general overview of the total population by analysing clinical data from the laboratory information system (LIS) and, from this, filtering to uncover the distribution of the reference population and the reference intervals. This approach has several advantages, since ‘big’ analytical data is more accessible nowadays [4]. Automation has increased in clinical laboratories. This has resulted in the centralization of medical tests from a big geographical area around Vall d’Hebron into a single LIS, which guarantees a common diagnostic test process and a similar data structure for extraction [6].

As a result of differences between reference intervals provided by different manufacturers and individual efforts to verify or select them from the literature, reference intervals vary per laboratory potentially resulting in unequal treatment and patient harm [7]. Standardization and harmonization efforts, which are currently successfully employed in several countries, are necessary to improve presentation and interpretation of laboratory results [8–14]. In the Netherlands, we previously determined nationally standardized reference intervals for clinical chemistry tests using an indirect “big data” approach [14]. A simple and straightforward workflow using the same approach is presented in this work. First, we determined indirect reference intervals using the NUMBER approach in a dataset of routine clinical chemistry values of the Vall d’Hebron laboratory population in Barcelona. The clinical laboratory Vall d’Hebron is the result of a fusion between three laboratories of the Catalan Institute of Health in Barcelona in 2014. It processes between 15,000 and 18,000 samples a day and covers a population of 1.2 million people, resulting in a very large amount of medical test results a year. This provided us with a unique opportunity to use only the data of a single laboratory using one single method to establish reference intervals, which is very important given the lack of harmonization in Spain [15]. Secondly, for those tests that are internationally standardized and produce test results traceable to standards and/or methods of higher order, we compared the reference intervals obtained from this study with the results published in the first NUMBER project in the Netherlands [14]. Finally, the reference intervals for creatinine kinase and uric acid were investigated, since no consensus was obtained yet in the NUMBER project [14].

## Material and methods

### Study design

We extracted anonymized medical test results from individuals visiting general practitioners, analysed from January 1^st^ 2018 until and including 31^st^ of December 2018 in the Clinical Laboratory Vall d’Hebron from the LIS. The presented study was considered suitable from the point of view of ethics and science by the corresponding Clinical Research Ethics Comittee.

We included test results from patients visiting primary care centres, employees analytical control centres, sexual and reproduction centres and geriatric centres. Test results were excluded when phlebotomy was performed in the hospital (inpatients), drug addiction centres, mental health centres, external emergency centres, the prison women centre, or at home (e.g. when primary care patients could not visit the laboratory due to illness) since we expected substantial differences in health status in these settings that can add noise to the data [4]. We performed sensitivity analyses to compare the distribution between all the included centres, showing no signs of sample or sex bias between centres (results not shown).

### Pre-analytical and analytical considerations

Samples were collected from 62 blood collection centres and were transported via 8 different routes to the laboratory. Serum tubes for biochemistry tests included separating gel and coagulation activator (BD Vacutainer^®^). Phlebotomy order of draw was always performed as advised by the EFLM pre-analytical workgroup [16]. The samples were transported to the laboratory in cool boxes with a temperature monitoring system. After arriving in the laboratory, the samples were centrifuged either 12 minutes at 3,500 rpm (2,438 g) when handled manually or 10 min at 3,000 rpm (2,113 g) when on the track.

Eighteen biochemistry tests were measured on three parallel AU5800 chemistry analysers (Beckman Coulter^®^). Detailed descriptions of the methods and the recommended reference intervals (calculated by direct approaches) according to Beckman’s IFU are presented in S1 Table. Tests included: albumin (CRM470 traceable), calcium (NIST-SRM-909bL1 traceable), creatinine (NIST-SRM-967 L1 traceable), lactate dehydrogenase (LDH) (not traceable to higher order reference material (NTRM)), magnesium (NIST-SRM-909bL2 traceable), anorganic phosphate (NTRM), total bilirubin (NIST-SRM-916a traceable), total protein (NIST-SRM-927c traceable), uric acid (traceable to isotope dilution Mass Spectrometry), urea (NIST-SRM-909bL1 traceable), chloride (NIST-SRM-919 traceable), potassium (NIST-SRM-918 traceable), sodium (NIST SRM-919 traceable), alkaline phosphatase (ALP) (NTRM), alanine aminotransferase (ALT) (NTRM), aspartate aminotransferase (AST) (NTRM), gamma glutamyltransferase (GGT) (traceable to IFCC reference method) and creatine kinase (CK) (traceable to IFCC reference method).

### Analytical quality assurance

To assure the outpatient data quality, we first examined the monthly results from external quality control scheme from the Spanish Society of Clinical Chemistry (SEQC), basic biochemistry scheme. In this scheme, the results obtained in our laboratory are compared with the average calculated from every laboratory participating in the program using the same analytical method and/or instrument. When our result was within one time the standard deviation from other laboratories participating in the scheme using the same method, data from this particular month and test were accepted as valid. When our result was above or below three standard deviations, we excluded the data from that particular test and instrument for that month. When the result was between the second and third standard deviation, we analysed the daily average outpatient results for the particular test and month.

Daily averages were investigated to ensure longitudinal accuracy of the results over time. Averages were calculated per batch of 200 results a day and were compared with the average per month and year. Plots were visually inspected in order to decide whether the analytical quality was sufficient using the biological variation of the monthly and yearly mean as a reference and comparing it visually with the daily mean.

Finally, due to the lack of commutable trueness verifiers in 2018, we further validated the quality of the obtained reference intervals by using a new data extraction of test results from 2020. In 2020, our laboratory participated in the fortnightly EQA scheme from the Dutch EQA organizer Stichting Kwaliteitsbewaking Medische Laboratoriumdiagnostiek (SKML) which uses commutable and value-assigned trueness verifiers [17]. In all EQA reports, the Multi sample evaluation (MUSE) scores for all tests were > = 1 (meaning a total allowable error sigma value over 2), indicating adequate performance for all tests [18]. To verify the calculated reference intervals deduced from the 2018 data, outpatient data from July to October 2020 were selected, considering the same analytical and pre-analytical considerations explained previously for the main data. To that end, we designed an algorithm that computed 2,000 random samples of 200 test results each time. Next, for each random sample of 200 test results, we calculated the proportion of cases residing within the reference intervals deduced from the 2018 dataset. Then we calculated the mean of these 2000 proportions for each test. When the mean of the proportions (Prop.2020) was higher than 95 %, we considered the reference interval as valid. This protocol was based on the CLSI EP28-A3C for reference intervals transference modifying the sample number from 20 to 200 and repeating the protocol 2,000 times [2].

### Clinical criteria

To avoid pre-analytical issues that could confound the reference intervals, results from hemolyzed, lipemic and icteric samples were excluded when indices were > = 2 on a 0–5 scale (Beckman Coulter^®^ AU5800, S2 Table). In addition, since the icteric index could also be a good indicator for liver dysfunction, samples with icteric indices > = 1 were also excluded for total bilirubin, ALT, AST, ALP and GGT.

For the calculations on CK, individuals with AST results higher than decision limits in Vall d’Hebron laboratory (50 U/L in men and 35 U/L in women) were excluded, in order to exclude patients with skeletal muscle injury [19].

### Statistical analyses

**Fig 1:**
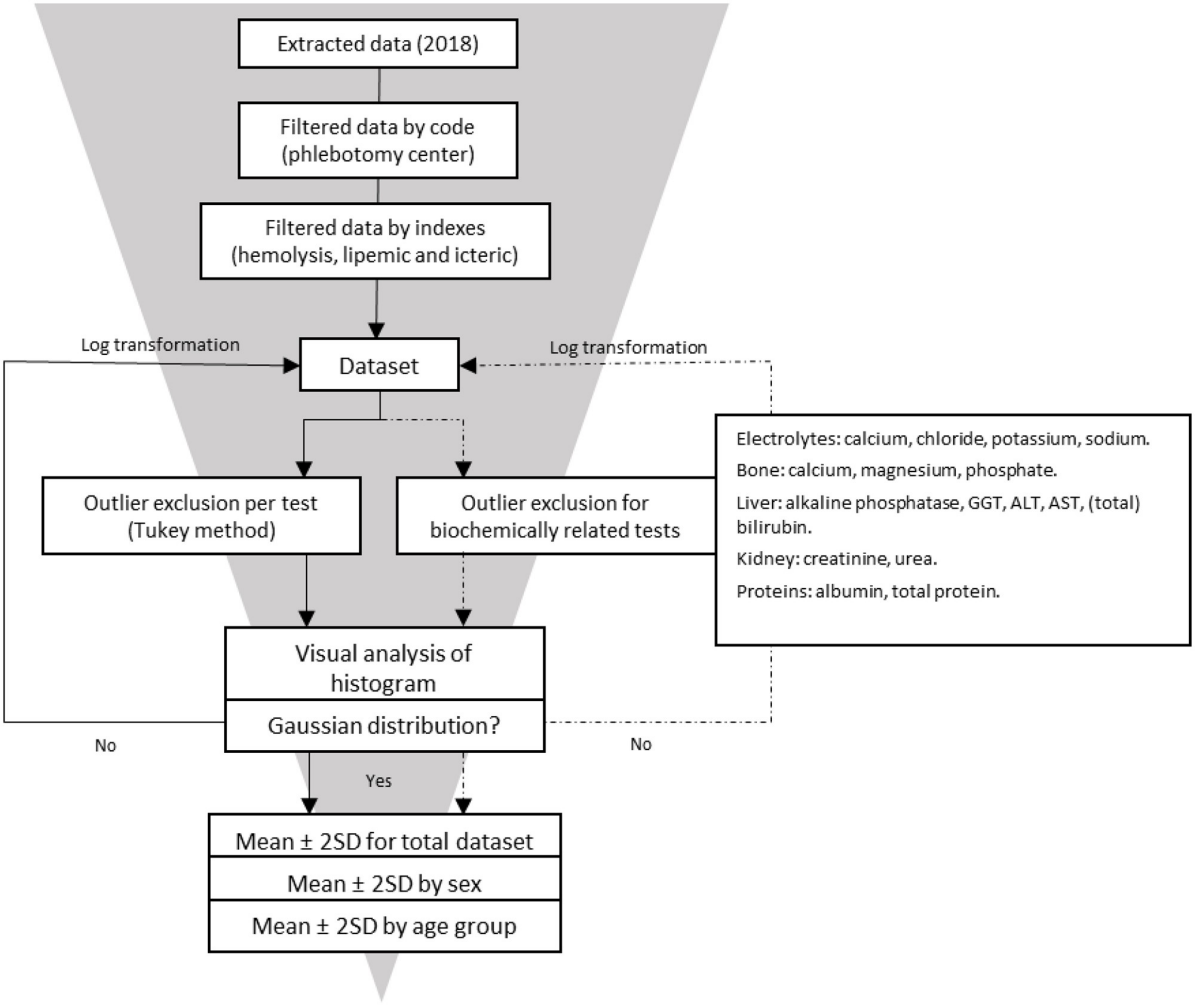
Study workflow. Workflow used for calculating reference intervals in Vall d’Hebron laboratory hospital by an indirect method based on the NUMBER study.

Reference intervals were calculated per test using an automatic calculator programmed in R [20] (version 3.6.1), following the workflow presented in Fig 1.

Firstly, we used the Tukey method [21] to identify and discard outliers. The lower and upper cut-offs for outlier exclusion were defined as Q1-(1.5xIQR) and Q3+(1.5xIQR), respectively, being Q1 the lower sample quartile, Q3 the upper sample quartile and IQR the interquartile range (Q3-Q1). The same workflow and outlier exclusion procedures were used as the ones described in the NUMBER project [14], where outliers from biochemically related tests based on defined groups were excluded. Defined groups were:

- Electrolytes: calcium, chloride, potassium, sodium
- Bone: calcium, magnesium, phosphate
- Liver: alkaline phosphatase, GGT, ALT, AST, (total) bilirubin
- Kidney: creatinine, urea
- Proteins: albumin, total protein

For calcium, two groups of tests were considered biochemically related. The histograms were visually inspected, and formal tests were performed (Z score for Skewness and Kurtosis) to determine the presence of a normal Gaussian distribution. Given the large numbers of test results, the formal tests of normality were very sensitive to a deviation from normality [22]. In such cases, visual inspection was considered decisive. If a normal distribution was absent, we performed a log transformation on the original data.

The reference intervals were calculated as mean plus/minus two times the standard deviation (mean ± 2SD) both for the total dataset and per subgroup when a minimum of 120 test results per group were available. Also 90% confidence intervals for the lower and upper limit were calculated. We used pre-defined subgroups analogous to the NUMBER project [14]:

- Sex: Male / Female
- Age:
  - Newborns /infants: <28 days of age (WHO definition), 28 days to <1 year
  - 1–5, 6–12, 13–18, 19–50, 51–65, 66–80, 80+ years

In addition, in order to test a recently published hypothesis [23] stating that certain differences between indirect studies may be due to diverse age representations into the age groups, sensitivity analyses with additional age categories were performed for ALT and GGT.

Per test and per group boxplots were visually inspected after outlier elimination in order to decide whether or not subgroup differentiated reference intervals were necessary. In addition, reference intervals results were compared with the reference limit estimator method employed by the group of Haeckel, Wosniok and Arzideh [24] using the same dataset.

Lastly, flagging rates were calculated to verify the clinical suitability of the reference intervals using an independent dataset (January–June 2019). The percentages of measurements below and above the lower and upper reference limits were calculated per test.

## Results

We extracted anonymized test results from a total of 530,778 clinical requests for a period of one year from the laboratory system of the Clinical Laboratory Vall d’Hebron University Hospital. After filtering by phlebotomy centre, 3.01% clinical requests were excluded. We discarded an additional 0.70% of the clinical requests because of hemolysis, 0.02% because of icteria, and 0.35% because of lipemia. The final dataset consisted of 509,408 requests.

Analytical performance, based on monthly external quality controls was adequate for SEQC material for all tests, except for ALP in December 2018. For this period, ALP results were excluded from the analyses. Daily average results showed stable performance over the year for all tests. In the S1 Fig we show an example for calcium.

Outlier exclusion by biochemically related tests ranged from 1.27 to 16.50%. Albumin, total protein, magnesium, phosphate, calcium, sodium, potassium and chloride followed a Gaussian distribution; for all other tests we obtained a Gaussian distribution after log transformation. The calculated reference intervals by the indirect approach are presented in Table 1, stratified for sex and age categories, if necessary. Results from the reference interval quality verification protocol, tested with the new dataset from 2020 (110,237 clinical requests), are also presented in Table 1, showing acceptable results (>95%) for all tests except for some age groups, particularly for creatinine and magnesium. Confidence intervals for the lower and upper limits per test are presented in S3 Table.

**Table 1:** Obtained Vall d’Hebron reference intervals results using the indirect approach from the NUMBER project, stratified for sex and age categories when necessary.

Test	Unit	Gender	Age, years	n	Calculated reference intervals:		
					Low	High	Prop. 2020*
Albumin	g/dL (g/L)	M	1–5	330	3.8 (38)	4.9 (49)	95.6
			6–18	914	4.1 (41)	5.0 (50)	92.8
			19–50	4281	4.0 (40)	5.1 (51)	89.4
			51–65	3660	3.8 (38)	4.9 (49)	96.2
			66–80	4704	3.5 (35)	4.9 (49)	98.6
			80+	4299	3.2 (32)	4.7 (47)	99.0
		F	1–5	282	3.9 (39)	4.9 (49)	93.2
			6–18	1116	4.0 (40)	5.0 (50)	94.5
			19–50	6622	3.7 (37)	4.9 (49)	94.0
			51–65	5966	3.8 (38)	4.8 (48)	93.4
			66–80	7865	3.6 (36)	4.7 (47)	93.6
			80+	10767	3.2 (32)	4.6 (46)	97.2
ALP	U/L	M	13–18	381	74	218	73.9
			19–50	11577	46	133	89.2
			51–65	9928	45	135	96.2
			66–80	9520	44	137	96.2
			80+	4569	46	155	94.5
		F	13–18	880	50	184	97.9
			19–50	15757	39	130	96.3
			51–65	14428	49	152	93.2
			66–80	15285	47	147	94.5
			80+	10185	46	157	94.4
ALT	U/L	M	1–12	2391	9	32	97.6
			13–18	2978	8	38	92.8
			19–50	45779	10	55	86.3
			51–65	37916	11	51	92.5
			66–80	42390	9	43	95.6
			80+	21014	7	34	92.0
		F	1–12	2308	9	31	97.5
			13–18	4748	7	27	95.4
			19–50	77714	7	35	95.7
			51–65	52710	9	42	97.2
			66–80	60878	8	36	97.2
			80+	4353	6	29	95.5
AST	U/L	M	1–5	557	25	51	99.5
			6–12	921	20	42	92.6
			13–18	1262	15	38	91.6
			19+	50953	13	38	96.1
		F	1–5	407	26	51	99.5
			6–12	1035	18	42	96.7
			13–18	1980	13	30	93.8
			19+	78383	13	36	95.9
Bilirubin (total)	mg/dL (μmol/L)	M	6–12	123	0.23 (4)	0.84 (14)	NA
			13–18	301	0.29 (5)	1.34 (23)	97.3
			19+	16323	0.32 (6)	1.30 (22)	95.1
		F	6–18	573	0.23 (4)	1.10 (19)	93.9
			19+	24215	0.28 (5)	1.04 (18)	95.5
Calcium	mg/dL (mmol/L)	M + F	1–5	318	9.2 (2.29)	10.7 (2.67)	NA
			6–12	954	9.3 (2.32)	10.5 (2.63)	97.7
			13–18	1358	9.2 (2.29)	10.5 (2.61)	95.4
			19+	46602	8.8 (2.20)	10.3 (2.58)	93.6
Chloride	mmol/L	M + F		784	98	108	91.1
Creatinine	mg/dL (μmol/L)	M	6–12	1317	0.37 (33)	0.61 (54)	68.1
			13–18	3517	0.47 (41)	1.07 (94)	93.0
			19–50	53345	0.65 (57)	1.17 (103)	66.3
			51–65	44666	0.62 (54)	1.23 (109)	78.8
			66–80	48705	0.62 (55)	1.36 (121)	83.1
			80+	22032	0.63 (56)	1.53 (135)	85.5
		F	6–12	1364	0.37 (33)	0.59 (53)	67.5
			13–18	4804	0.45 (40)	0.83 (74)	86.6
			19–50	80957	0.47 (41)	0.90 (79)	82.1
			51–65	56832	0.47 (41)	0.95 (84)	85.5
			66–80	67350	0.46 (41)	1.09 (96)	90.7
			80+	47750	0.48 (42)	1.37 (121)	97.8
GGT	U/L	M	1–5	174	7	20	NA
			6–12	322	9	23	97.2
			13–18	1452	8	36	95.5
			19–50	31582	9	79	96.0
			51–65	26758	12	95	93.9
			66–80	28080	11	84	96.5
			80+	13160	8	79	99.1
		F	1–5	146	8	17	NA
			6–12	341	8	22	95.7
			13–18	2191	7	26	97.2
			19–50	48040	7	48	99.0
			51–65	35997	8	71	99.7
			66–80	40412	8	65	100
			80+	27174	7	66	100
LDH	U/L	M + F	6–12	257	359	643	NA
			13–18	340	274	531	NA
			19–50	2573	256	507	NA
			51–65	1963	274	534	NA
			66–80	2039	270	551	NA
			80+	1539	266	584	NA
Magnesium	mg/dL (mmol/L)	M + F		4571	1.8 (0.72)	2.4 (1.00)	89.5
Phosphate	mg/dL (mmol/L)	M	1–5	147	4.2 (1.34)	5.4 (1.74)	NA
			6–12	405	4.2 (1.33)	5.3 (1.7)	94.8
			13–18	390	3.6 (1.16)	5.4 (1.72)	94.4
			19–50	3132	2.4 (0.77)	4.7 (1.51)	96.8
			51–65	2917	2.2 (0.72)	4.3 (1.38)	90.0
			66+	6640	2.2 (0.71)	4.2 (1.34)	92.7
		F	1–5	126	4.3 (1.39)	5.4 (1.71)	96.8
			6–12	428	4.1 (1.32)	5.3 (1.71)	96.3
			13–18	729	3.5 (1.11)	5.2 (1.67)	94.5
			19–50	5874	2.6 (0.84)	4.7 (1.51)	93.0
			51–65	7970	2.7 (0.88)	4.7 (1.5)	92.8
			66+	18548	2.6 (0.84)	4.5 (1.43)	93.9
Potassium	mmol/L	M + F		257189	3.60	5.09	95.4
Sodium	mmol/L	M + F		256775	136	144	95.7
Total protein	g/dL (g/L)	M + F		35141	6.1 (61)	8.0 (80)	94.8
Urea	mg/dL (mmol/L)	M	1–5	227	15 (2.5)	45 (7.5)	93.4
			6–12	755	19 (3.1)	47 (7.7)	88.7
			13–18	996	18 (3.0)	47 (7.8)	85.7
			19–50	4709	20 (3.3)	54 (9.0)	86.1
			51–65	4457	21 (3.5)	61 (10.2)	93.9
			66–80	5680	23 (3.9)	75 (12.6)	95.6
			80+	3662	27 (4.5)	93 (15.5)	94.5
		F	1–5	167	16 (2.6)	43 (7.2)	91.0
			6–12	767	17 (2.8)	44 (7.4)	92.9
			13–18	1220	16 (2.7)	42 (7.0)	92.0
			19–50	6850	16 (2.7)	46 (7.7)	93.9
			51–65	5524	20 (3.4)	58 (9.7)	94.8
			66–80	6986	22 (3.7)	72 (12.0)	96.4
			80+	7668	25 (4.1)	97 (16.2)	96.1

Obtained Vall d’Hebron reference intervals using the indirect approach from the NUMBER project stratified by sex and age categories when necessary.

M: Male, F: Female.

^a^The reference intervals obtained from the dataset in 2018 were validated using a new dataset in 2020 when the laboratory participated in a type 1 EQA scheme. Proportion (Prop.) 2020 indicates the proportion of data from 2020 inside the calculated reference intervals. When the mean of the proportions was higher than 95%, we considered the calculated reference intervals verified.

**Fig 2:**
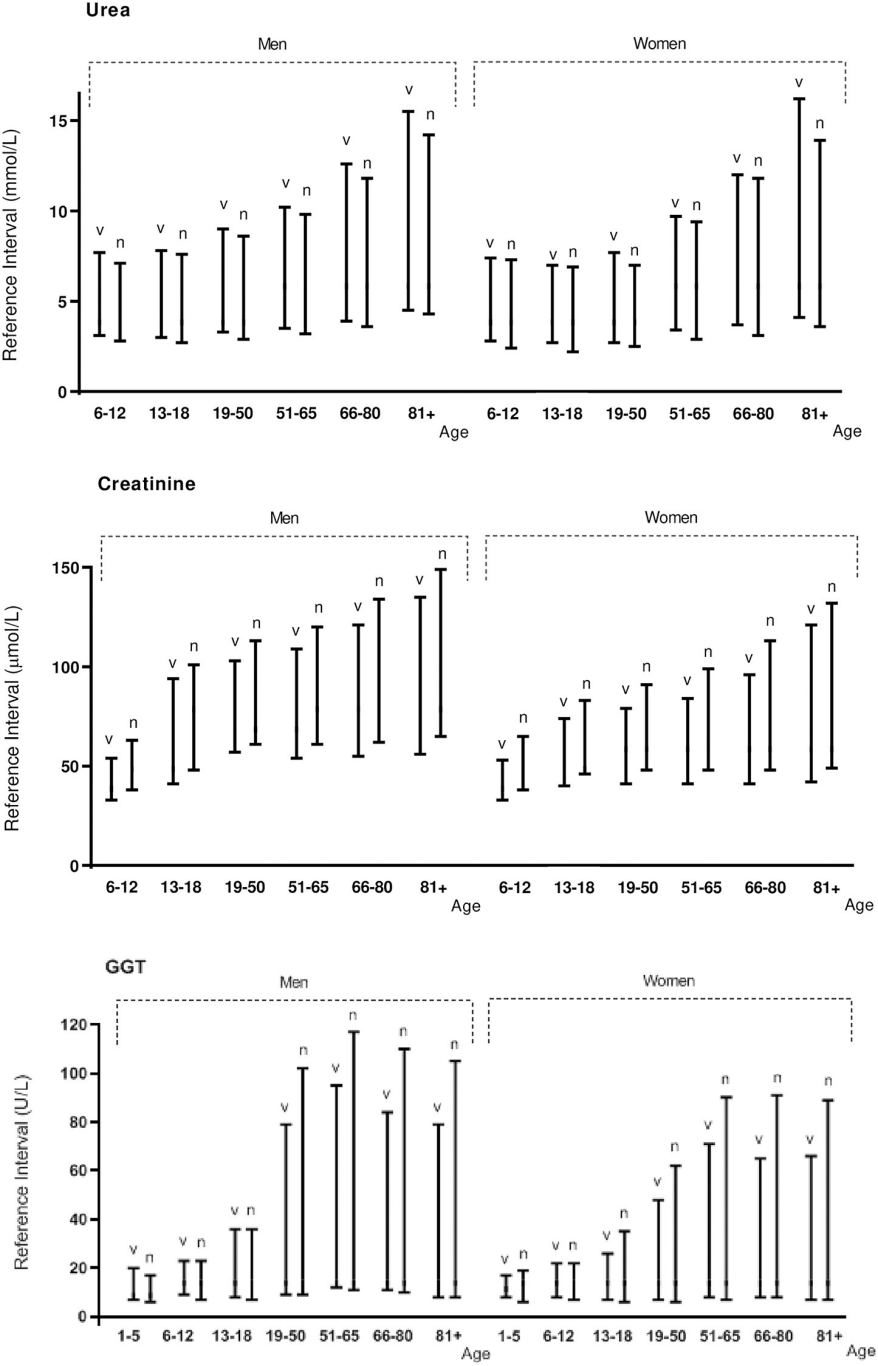
Urea, creatinine and GGT results. Age and sex effects on the reference intervals for creatinine, urea and GGT for Vall d’Hebron (v) and NUMBER (n).

**Fig 3:**
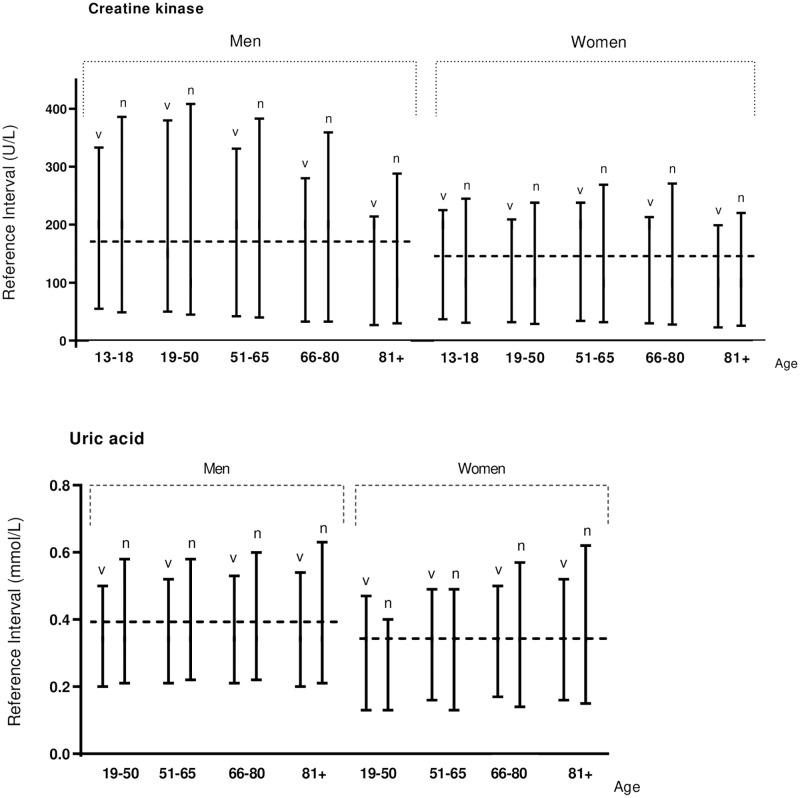
Creatine kinase and uric acid results. Reference intervals for creatine kinase and uric acid for Vall d’Hebron (v) and NUMBER (n), stratified for age and sex. Currently used upper reference interval in Vall d’Hebron are shown as slashed lines.

In Table 2, the obtained Vall d’Hebron reference intervals from the normally distributed tests are compared with results from the Dutch NUMBER project [14]. The kidney and liver parameters for both studies are graphically displayed in different age categories for men and women in Fig 2. Similar results for GGT were found when we increased the number of age categories (S2 Fig). In addition, results from the calculated reference intervals for creatine kinase and uric acid for the Vall d’Hebron hospital and the Dutch project are presented in Fig 3.

**Table 2:** Reference intervals results from normally distributed tests.

Test	Unit	Gender	Age, years	Vall d’hebron RI:		NUMBER RI:	
				Low	High	Low	High
Albumin	g/dL (g/L)	M	6–18	4.2 (42)	5.1 (51)	4.0 (40)	5.2 (52)
			19–50	4.0 (40)	5.1 (51)	3.9 (39)	5.1 (51)
			51–65	3.8 (38)	4.9 (49)	3.7 (37)	4.9 (49)
			66–80	3.5 (35)	4.9 (49)	3.6 (36)	4.8 (48)
			80+	3.2 (32)	4.7 (47)	3.6 (36)	4.6 (46)
		F	1–5	4.0 (40)	5.0 (50)	3.9 (39)	5.0 (50)
			6–18	4.0 (40)	5.1 (51)	4.0 (40)	5.1 (51)
			19–50	3.7 (37)	4.9 (49)	3.8 (38)	4.9 (49)
			51–65	3.8 (38)	4.8 (48)	3.8 (38)	4.9 (49)
			66–80	3.6 (36)	4.7 (47)	3.7 (37)	4.8 (48)
			80+	3.2 (32)	4.6 (46)	3.6 (36)	4.7 (47)
Calcium	mg/dL (mmol/L)	M + F	6–12	9.3 (2.32)	10.5 (2.63)	9.2 (2.29)	10.3 (2.56)
			13–18	9.2 (2.29)	10.5 (2.61)	8.9 (2.23)	10.3 (2.57)
			19+	8.8 (2.20)	10.3 (2.58)	8.7 (2.18)	10.2 (2.55)
Chloride	mmol/L	M + F		98	108	97	108
Phosphate	mg/dL (mmol/L)	M	13–18	3.6 (1.16)	5.4 (1.72)	2.9 (0.88)	4.8 (1.53)
			19–50	2.4 (0.77)	4.7 (1.51)	1.9 (0.62)	4.1 (1.32)
			51–65	2.3 (0.72)	4.3 (1.38)	1.9 (0.62)	4.1 (1.32)
			66+	2.2 (0.71)	4.2 (1.34)	1.9 (0.62)	4.1 (1.32)
		F	13–18	3.5 (1.11)	5.2 (1.67)	2.6 (0.82)	4.8 (1.52)
			19–50	2.6 (0.84)	4.7 (1.51)	2.3 (0.73)	4.5 (1.44)
			51–65	2.8 (0.88)	4.7 (1.50)	2.3 (0.73)	4.5 (1.44)
			66+	2.6 (0.84)	4.5 (1.43)	2.3 (0.73)	4.5 (1.44)
Potassium	mmol/L	M + F		3.6	5.1	3.8	5.2
Magnesium	mg/dL (mmol/L)	M + F		1.75 (0.72)	2.43 (1.00)	1.73 (0.71)	2.38 (0.98)
Sodium	mmol/L	M + F		136	144	136	145
Total protein	g/dL (g/L)	M + F		6.1 (61)	8.0 (80)	6.1 (61)	7.9 (79)

The obtained Vall d’Hebron reference intervals for the normally distributed tests, compared with results from the Dutch NUMBER project, stratified for sex and age categories, if necessary.

Results calculated using the reference limit estimator method are presented in S4 Table and S3 Fig.

**Fig 4:**
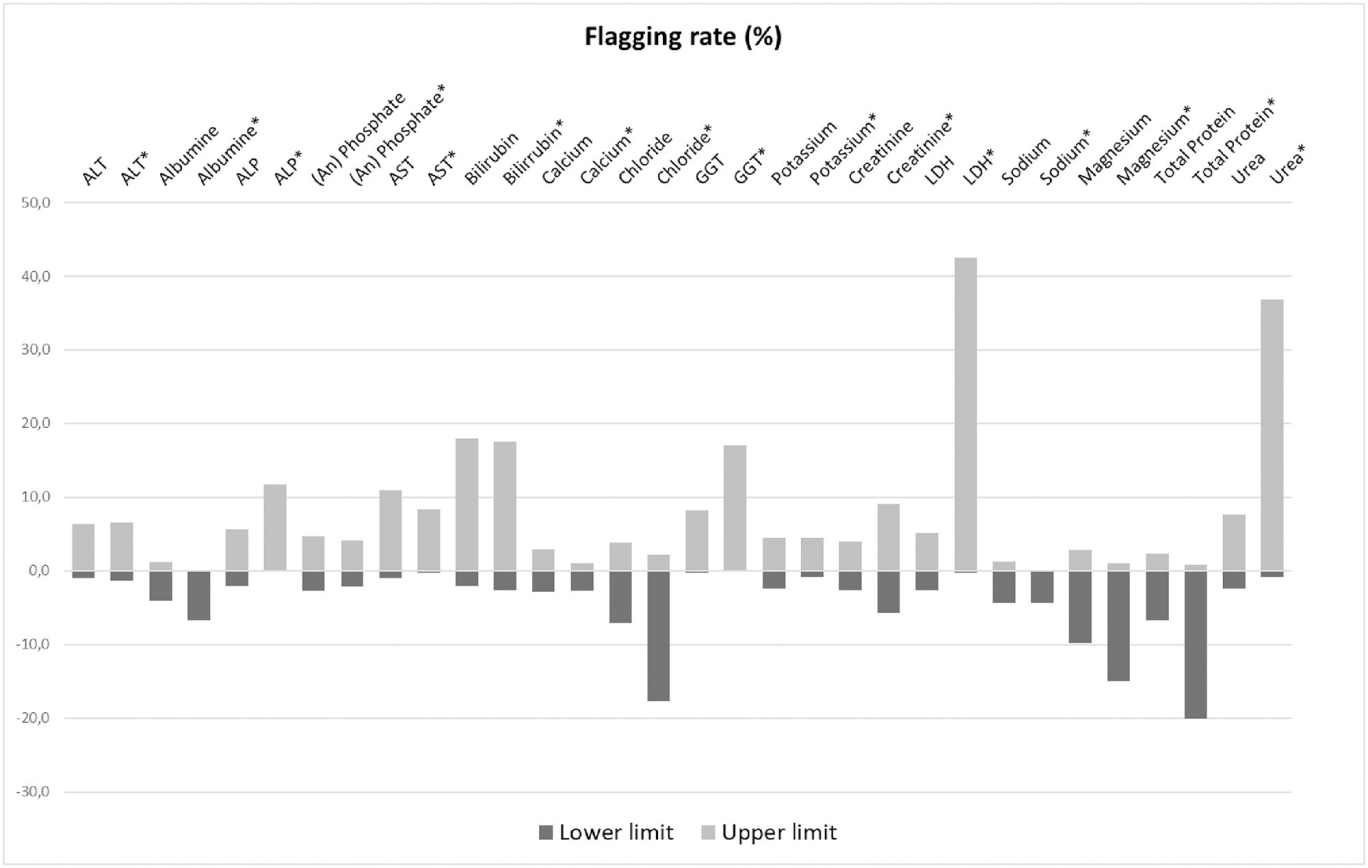
Flagging rates. Percentage of individuals upper or lower (represented as negative) the reference intervals, for an independent dataset (January-June 2019) for both calculated reference intervals and currently used reference intervals in Vall d’Hebron (*).

Flagging rates from an independent dataset, for both the calculated reference intervals in this study and the currently used reference intervals in Vall d’Hebron laboratory are presented in Fig 4.

## Discussion

Application of big data to healthcare has been a matter of interest in recent years [25]. Consequently, in laboratory medicine, where quantitative data is generated every day, machine learning, data mining, business intelligence and related concepts are starting to be used for different purposes including analytical and quality management [25]. For the determination of reference intervals, for which classical (direct) recommendations are laborious and expensive, various statistical (indirect) methods have been developed using big data [4]. It is important to remark that some specialists are concerned about the possible bias due to the presence of unhealthy individuals in the dataset. Standard and detailed protocols following this approach are not available yet. However, the IFCC committee on Reference Intervals and Decision Limits (c-RIDL) recently recommended and promoted the development and assessment of indirect methods, stimulating future consensus for a harmonized indirect approach [26].

In the present study, we calculated reference intervals in an outpatient population from Vall d’Hebron laboratory using the NUMBER approach created for calculating nationally standardized reference intervals for clinical chemistry tests in The Netherlands [14]. The normally distributed tests (Table 2) showed similar reference intervals between both studies and other previous projects such as the Canadian project CALIPER (direct method) [27], the Australian and New Zeeland project ARIA (direct method) [8], or the German projects (indirect methods) [23,24]. This suggests that standardized tests allow global and common use of reference intervals and a straightforward indirect method could be a valuable approach for these normally distributed tests. The comparison of the results from this study with the reference limit estimator method (S4 Table and S3 Fig) support this idea as nearly equal reference interval calculations were obtained with both methods for tests with a normal distribution.

In this project, the upper reference limits for liver enzymes from the Dutch project were always substantially higher than the upper reference limits from Vall d’Hebron laboratory. We previously already hypothesized about potential explanations for the higher upper limits in the Netherlands [14], as a result of the Dutch lifestyle and diet. The only IFCC-standardized method for liver parameters in our study was GGT and the differences for this test between Vall d’Hebron results in Barcelona and the NUMBER project could support this hypothesis.

Alcohol consumption and increased body mass index have been related with higher ALT, GGT and AST results in the population from the Nordic Reference Interval Project (NORIP) [28]. Interestingly, in 2009, Strømme and colleagues, using data from the NORIP project, showed reference intervals results for ALT in northern Europe which are similar to our Dutch results [29]. They already highlighted the differences observed between the Nordic reference intervals and the reference intervals calculated for the Italian population, which in their turn are similar to the calculated reference intervals in our study for the population in Vall d’Hebron [29,30]. In a recent publication, Wosniok et al. addressed these differences in calculated reference intervals from different studies for liver parameters [23]. They proposed it may be due to diverse age representations in the age groups. In order to test this hypothesis, we repeated the analyses for GGT, applying more age categories, in both the Vall d’Hebron and NUMBER datasets (S2 Fig). Since these results showed the same tendency, we consider differences in lifestyle a potential alternative hypothesis. In addition, the reference intervals for GGT are only significantly higher in the adult Dutch population (when diet or alcohol do start to play a role) and not in children, indicating a lifestyle component. The Mediterranean diet has been associated with favourable health outcomes [31], and with decreasing levels of ALT, AST and GGT in patients with non-alcoholic fatty liver disease, supporting this hypothesis [32]. It is important to remark that the reference intervals for the liver parameters that were calculated using the reference limit estimator method (S4 Table and S3 Fig) were not as high in the Vall d’Hebron population as with NUMBER method, but were still higher than the reference intervals that are now commonly applied in clinical laboratories. This supports the idea that, for skewed distributions, it is still necessary to further explore the best indirect method for references interval calculation.

For creatinine and urea, similar age distributions were found in the Vall d’Hebron outpatient sample compared to the Dutch national sample, even though the methodology for creatinine differed (Jaffe vs enzymatic, Fig 2), which support earlier studies on the age related decline in renal function [33].

Interestingly, for reasons yet unclear, in age group 19–50 years, for albumin, ALP, ALT, creatinine and urea, the resulting reference interval is usually smaller in male patients and the Prop. 2020 is always lower (<90%) when comparing to the results in female patients. No explanation was found for the significantly elevated reference intervals for CK and uric acid in the NUMBER project [14], as the calculated reference intervals were substantially higher than those currently applied in the participating laboratories. In the Vall d’Hebron sample, we confirmed the Dutch observations and also found reference intervals higher than currently used and recommended for these tests. Nevertheless, compared with the Duch results, the upper limits of the reference intervals calculated in Vall d’Hebron laboratory were lower for all age groups for both CK and uric acid (Fig 3). For CK, differences between currently used and calculated reference intervals are particularly extreme, which has been already observed in other studies [34,35]. This finding might be explained by the high incidence of some related comorbidities such as metabolic syndrome or high blood pressure [36] together with the use of statins. For uric acid, the obtained higher limits in both studies are also higher than cut-off values associated with worse progression of kidney disease [37] and higher than the cut-off defined by the solubility limit of uric acid [14].

Our analyses show important differences in flagging rates between the currently used reference intervals in Vall d’Hebron and the new calculated reference intervals in an independent dataset. In general terms, too much flagging is noted for currently used reference intervals. This highlights the need for establishing adequate reference intervals, as frequent flagging may distract attention from true pathological results [38]. In addition to that, we found, in general, higher flagging in our study compared to the Dutch NUMBER study which may be explained by the additional pre-analytical and clinical criteria used in the current study.

For some of the calculated reference intervals the confidence intervals for lower and upper limits (S3 Table) included only the reported limit, due to the large sample size, emphasizing the robustness of the presented results.

It is also important to remark that, in general, the results calculated with the NUMBER method and the Reference Limit Estimator method (S4 Table and S3 Fig) show in a great extent similar results across age group, but for a few laboratory tests there are some remarkable differences that deserve further study. Lower reference intervals were found with the Reference Limit Estimator method for GGT, creatinine and CK.

Our study has several strengths. First, compared to the direct method of establishing reference intervals, the applied automatic indirect approach is cost-efficient and avoids collecting and analysing material from healthy control donors. Second, it mimics preanalytical conditions of real samples. In addition, we had the unique opportunity to experiment with the Dutch NUMBER approach and to do head-to-head comparisons between the reference intervals obtained for the Dutch population with the reference intervals calculated in the Vall d’Hebron population for standardized tests. Lastly, results using the NUMBER method were also compared with the reference limit estimator method [24] using the same dataset.

We also acknowledge several limitations. First, since we used anonymous laboratory test results, clinical information was not available. Although we tried to select a healthy population as much as possible, test results from unhealthy persons may have been included in our datasets. Second, because of our completely anonymized databases, we did not exclude individuals visiting practitioners more than once a year leading to a possible bias. Third, structural monitoring with commutable, value-assigned trueness verifiers (type 1 EQA-materials) was not available in 2018. However, blinded type 1 EQA materials from the Dutch SKML were used in 2020, which is essential for proving metrological traceability of results from standardized test. By using a random sampling method with a dataset from 2020 we confirmed adequate analytical performance and verified the reference intervals calculated in the 2018 dataset. The COVID-19 pandemic and the resulting differences in patient population hampered us in using a dataset from 2020 to calculate the reference intervals. Fourth, we selected one statistical method (NUMBER method) to calculate reference intervals, and compared these with the reference limit estimator method [24]. Several statistical methods have been proposed so far but no consensus or official recommendations about ‘which method to use when’ are available yet [4]. We recommend that, on an international level, indirect (statistical) reference interval methods are compared, in order to reach consensus on criteria to decide which statistical method should be applied for which test. Given the comparable results between studies applying indirect methods to establish reference intervals, indirect methods are a promising tool for laboratories to develop cheap, specific and updated reference intervals.

In conclusion, using an indirect approach, we determined population-specific reference intervals for 16 biochemistry tests from the Vall d’Hebron region, some being more sex and age specific than in the product inserts. Reference intervals of normally distributed biochemical tests were comparable to those found in a Dutch outpatient sample, indicating that the indirect method is an appropriate approach for deducing reference intervals. In order to verify the applicability of SI-traceable reference intervals obtained by indirect methods across outpatient populations, equivalence of test results from SI-standardizable tests must be verified thoroughly using type 1 EQA-materials. To conclude, adequate implementation of common, metrologically traceable reference intervals is the ultimate goal for guaranteeing safe and clinically effective use of medical tests, as required by the upcoming EU IVD Regulation 2017/746. As a first step, method (Beckman)- and population (Vall d’Hebron region)- specific refined reference intervals were derived for biochemistry tests.

## Supporting information

S1 Fig Daily averages plot for calcium. Daily average is represented as points, monthly average as black lines and the average of the year as red lines. Slashed lines represent biological variation from monthly (black) or yearly (red) average and were used as an indication for person to person variation. Decisions about quality stability were made by visual inspection of the plots.
(PDF)

S2 Fig GGT reference interval results by age. Different age representation for the calculated reference intervals for ALT and GGT for Vall d’Hebron (V) and NUMBER (N).
(PDF)

S3 Fig Comparison between indirect reference intervals using two methods. NUMBER method and reference limit estimator (RLE) method. Representation of reference intervals from S4 Table were made just when the number of data per both methods were higher than 500. *Reference interval results calculated with less data than the recommended by the RLE method (4.000).
(PDF)

S1 Table Methods principles and metrological traceability of general clinical chemistry tests used in Vall d’Hebron for determining reference intervals. LOINC codes for international units are also shown in the table.
(PDF)

S2 Table Corresponding approximate serum concentrations of intralipid, bilirubin and free hemoglobin for the 0–5 scale for indices. (PDF)

S3 Table Calculated reference intervals using NUMBER method presented together with the 90% confidence interval. (PDF)

S4 Table Comparison of indirect reference intervals using the NUMBER method and the reference limit estimator (RLE) method. Confidence intervals are presented for the RLE method. Results are presented in calculated and international units.
(PDF)
