# Supplementary figures and images for: Indirect determination of biochemistry reference intervals using outpatient data
Source: PLoS One. 2022 May 19;17(5):e0268522. doi: 10.1371/journal.pone.0268522 (PMC9119462; doi:10.1371/journal.pone.0268522)

**S1 Fig.**

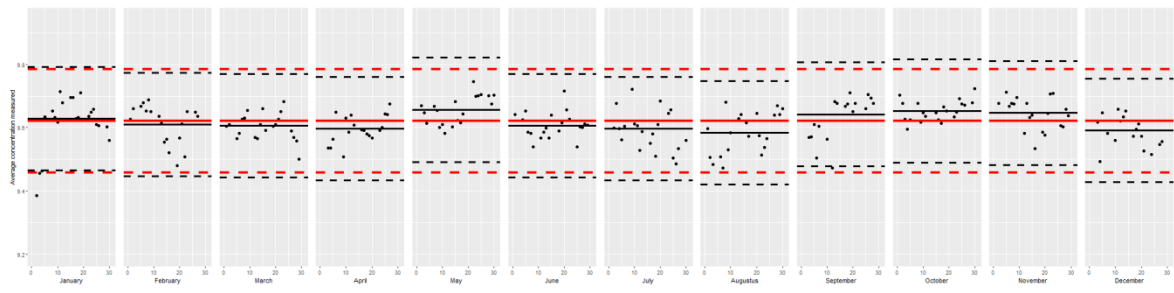

Supplement: S1 Fig — Daily average is represented as points, monthly average as black lines and the average of the year as red lines. Slashed lines represent biological variation from monthly (black) or yearly (red) average and were used as an indication for person to person variation. Decisions about quality stability were made by visual inspection of the plots. (PDF) [file pone.0268522.s001.pdf]

**S2 Fig.**

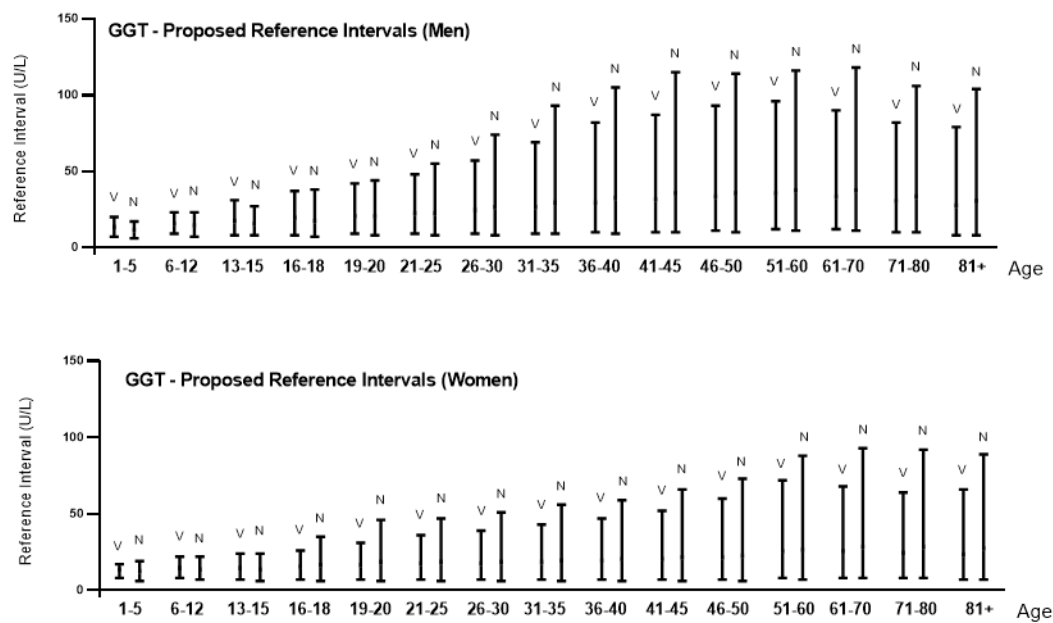

Supplement: S2 Fig — Different age representation for the calculated reference intervals for ALT and GGT for Vall d’Hebron (V) and NUMBER (N). (PDF) [file pone.0268522.s002.pdf]

S3 Fig.

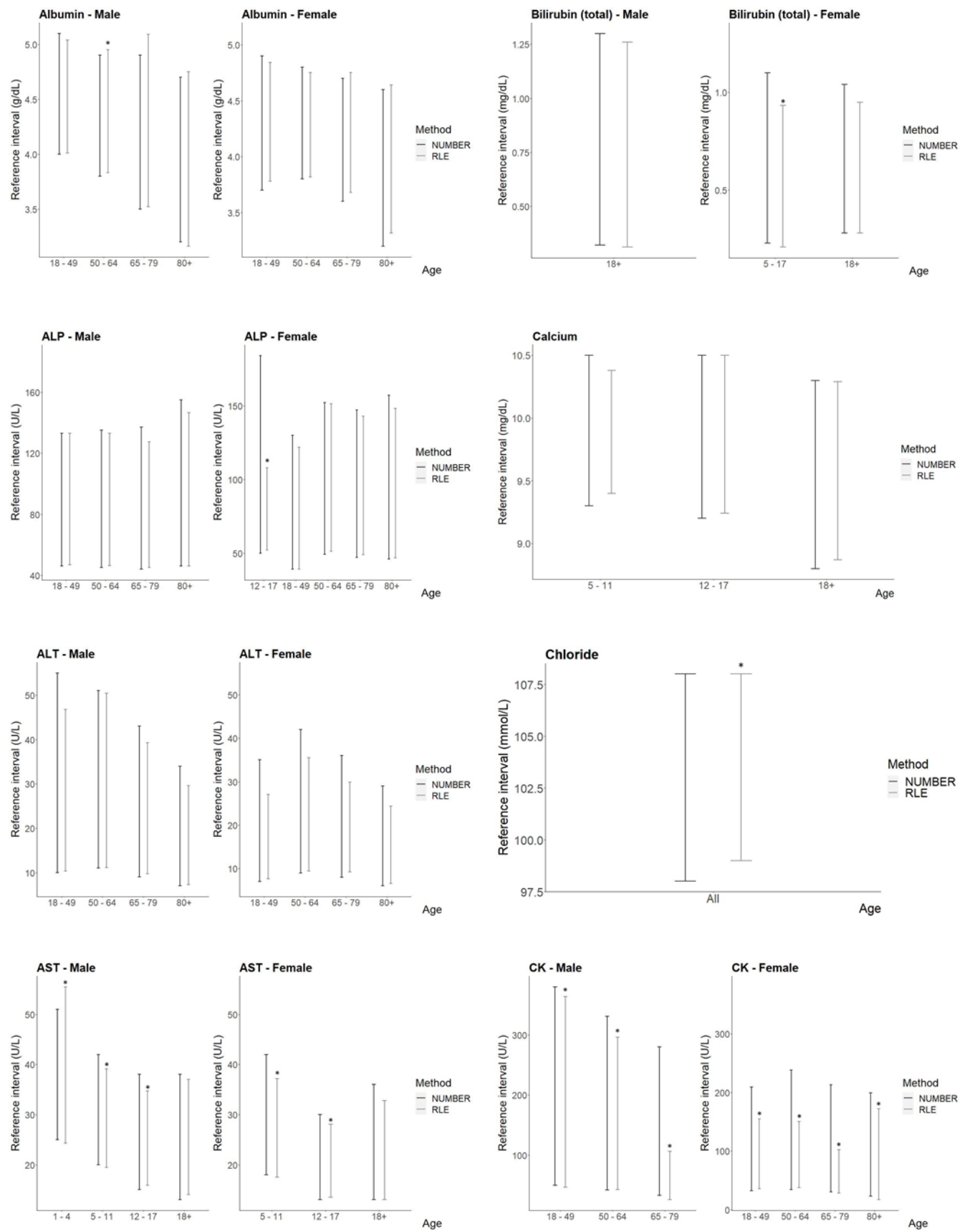

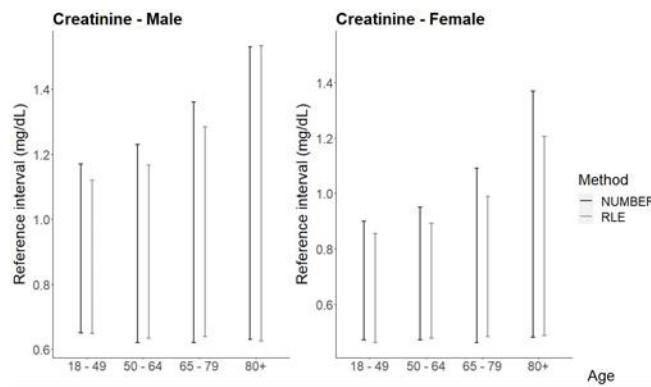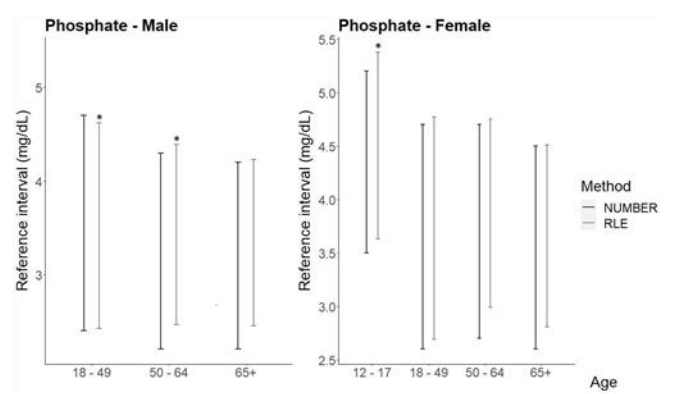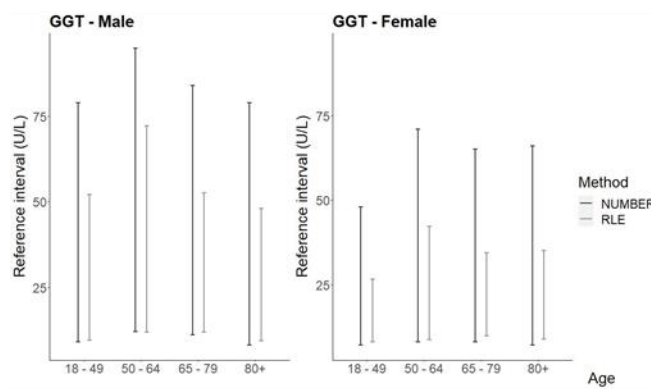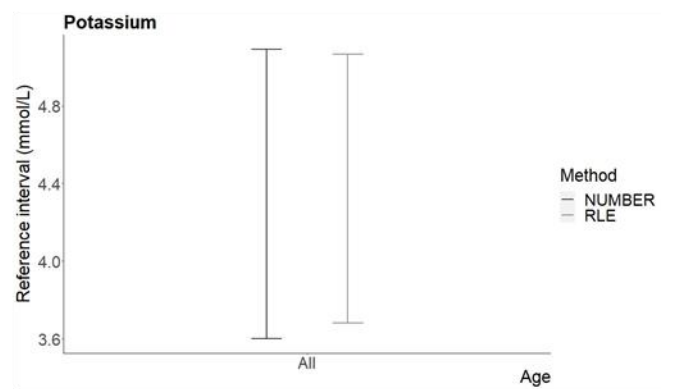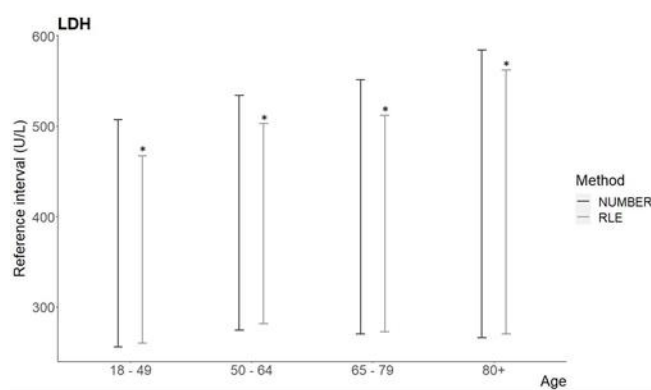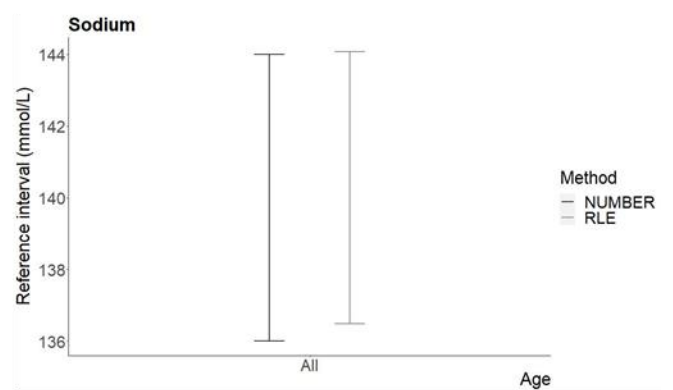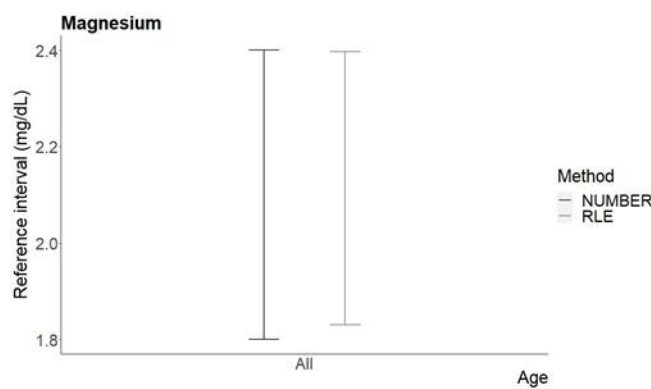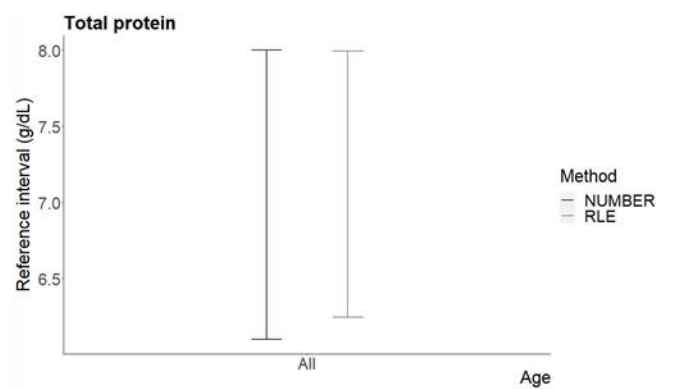

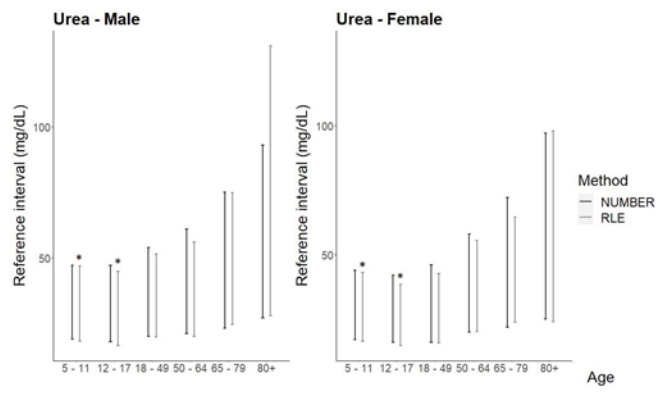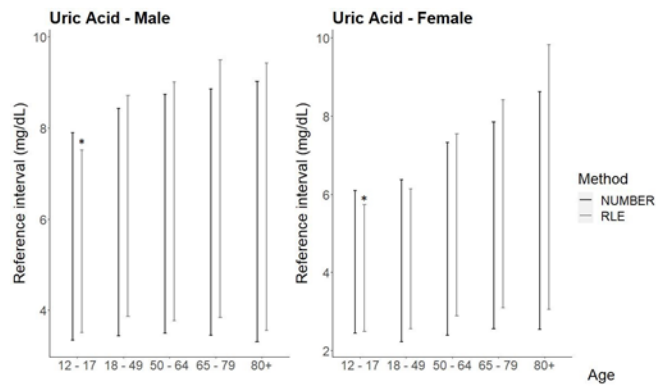

Supplement: S3 Fig — NUMBER method and reference limit estimator (RLE) method. Representation of reference intervals from S4 Table were made just when the number of data per both methods were higher than 500. *Reference interval results calculated with less data than the recommended by the RLE method (4.000). (PDF) [file pone.0268522.s003.pdf]
