# Supplementary material for: Indirect determination of biochemistry reference intervals using outpatient data
Source: PLoS One. 2022 May 19;17(5):e0268522. doi: 10.1371/journal.pone.0268522 (PMC9119462; doi:10.1371/journal.pone.0268522)
Supplement: S1 Table — LOINC codes for international units are also shown in the table. (PDF) [file pone.0268522.s004.pdf]

S1 Table.

Test (LOINC*)	Traceability	Method short description	Catalogue Number	Currently used and recommended reference intervals (adults)
Albumin (61151-7)	CRM 470	bromocesol green	OSR6202	35 – 52 g/L
Calcium (2000-8)	NIST SRM 909bL1	reaction with arsenazo III	OSR61117	2.20 – 2.65 mmol/L
Creatinine (14682-9)	NIST SRM 967 L1	Jaffé method IDMS traceable (compensated)	OSR6178	M: 59 – 104 µmol/L<br>F: 45 – 84 µmol/L
Lactate Dehydrogenase (14805-6)	Beckman Master Cal*	pyruvate to lactate method	OSR6126	208 – 378 U/L
Magnesium (2601-3)	NIST SRM 909b L2	direct method with xylidyl blue in basic reaction	OSR6189	M: 0.73 – 1.06 mmol/L<br>F: 0.77 – 1.03 mmol/L
(Anorganic) Phosphate (14879-1)	Beckman Master Cal*	reaction with molybdate	OSR6222	0.81 – 1.45 mmol/L
Total Bilirubin (14631-6)	NIST SRM 916a	reaction with 3,5-tetrafluoroborato de diclorofenilodiazonio stabilized by diazonium salt	OSR6212	5 – 21 µmol/L
Total Protein (2885-2)	NIST SRM 927c	copper reaction in basic solution (Biuret)	OSR6232	66 – 83 g/L
Uric Acid (14933-6)	Isotope Dilution Mass Spectrometry	uricase total reaction to form alantoine and hydrogen peroxide and coupled Trinder reaction for hydrogen peroxide determination	OSR6298	M : 208.3 – 428.4 µmol/L<br>F: 154.7 – 357.0 µmol/L
Urea (22664-7)	NIST SRM 909b L1	indirect method with urease reaction to form ammonium ion and carbonate and coupled reaction with glutamate dehydrogenase for ammonium determination	OSR6234	2.8 – 7.2 mmol/L
Chloride (2075-0)	NIST SRM 919	indirect ion selective electrodes	A28937 & A28945	98 – 107 mmol/L
Potassium (2823-3)	NIST SRM 918	indirect ion selective electrodes	A28937 & A28945	3.5 – 5.1 mmol/L
Sodium (2951-2)	NIST SRM 919	indirect ion selective electrodes	A28937 & A28945	136 – 145 mmol/L
Alkaline Phosphatase (6768-6)	Beckman Master Cal**	IFCC recommended method	OSR6004	M : 43 – 115 U/L<br>F: 33 – 98 U/L

Alanine Aminotransferase (1744-2)	Beckman Master Cal**	IFCC recommended method without pyridoxyl 5 phosphate	OSR6107	M: <50 U/L<br>F: <35 U/L
Aspartate Aminotransferase (88112-8)	Beckman Master Cal**	IFCC recommended method without pyridoxyl 5 phosphate	OSR6209	M: <50 U/L<br>F: <35 U/L
Gamma-Glutamyltransferase (2324-2)	IFCC reference method	IFCC recommended method	OSR6120	M: <55 U/L<br>F: <38 U/L
Creatine Kinase (2157-6)	IFCC reference method	IFCC recommended method	OSR6279	M: <171 U/L<br>F: <145 U/L

* LOINC codes are indicated for the units presented in the same table (international units)

** Beckman Coulter system calibrator catalogue number 66300 with values determined by Beckman Coulter selected measurement procedure.
