# Supplementary material for: Indirect determination of biochemistry reference intervals using outpatient data
Source: PLoS One. 2022 May 19;17(5):e0268522. doi: 10.1371/journal.pone.0268522 (PMC9119462; doi:10.1371/journal.pone.0268522)
Supplement: S2 Table — (PDF) [file pone.0268522.s005.pdf]

S2 Table.

Scale	Intralipid mg/dL	Bilirubin mg/ dL	Hemoglobin mg/dL
1	40 – 99	1.4 – 4.9	50-99
2	100-199	5.0 – 9.9	100-199
3	200-299	10.0 – 19.9	200-299
4	300-500	20 - 40	300-500
5	>500	> 40	>500
