# Supplementary material for: Indirect determination of biochemistry reference intervals using outpatient data
Source: PLoS One. 2022 May 19;17(5):e0268522. doi: 10.1371/journal.pone.0268522 (PMC9119462; doi:10.1371/journal.pone.0268522)
Supplement: S3 Table — (PDF) [file pone.0268522.s006.pdf]

S3 Table.

Test	Unit	Sex	Age. years	n	Calculated reference intervals: Low	90% CI	High	90% CI
Albumin	g/dL	M	1-5	330	3.8	(3.76-3.84)	4.9	(4.86-4.94)
			6-18	914	4.1	(4.08-4.12)	5.0	(4.98-5.02)
			19-50	4281	4.0	(3.99-4.01)	5.1	(5.09-5.11)
			51-65	3660	3.8	(3.79-3.81)	4.9	(4.89-4.91)
			66-80	4704	3.5	(3.49-3.51)	4.9	(4.89-4.91)
			80+	4299	3.2	(3.18-3.22)	4.7	(4.68-4.72)
		F	1-5	282	3.9	(3.86-3.94)	4.9	(4.86-4.94)
			6-18	1116	4.0	(3.98-4.02)	5.0	(4.98-5.02)
			19-50	6622	3.7	(3.69-3.71)	4.9	(4.89-4.91)
			51-65	5966	3.8	(3.79-3.81)	4.8	(4.79-4.81)
			66-80	7865	3.6	(3.59-3.61)	4.7	(4.69-4.71)
			80+	10767	3.2	(3.19-3.21)	4.6	(4.59-4.61)
ALP	U/L	M	13-18	381	74	(68.8-79.2)	218	(212.8-223.2)
			19-50	11577	46	(45.4-46.6)	133	(132.4-133.6)
			51-65	9928	45	(44.4-45.6)	135	(134.4-135.6)
			66-80	9520	44	(43.3-44.7)	137	(136.3-137.7)
			80+	4569	46	(44.9-47.1)	155	(153.9-156.1)
		F	13-18	880	50	(46.8-53.2)	184	(180.8-187.2)
			19-50	15757	39	(38.5-39.5)	130	(129.5-130.5)
			51-65	14428	49	(48.4-49.6)	152	(151.4-152.6)
			66-80	15285	47	(46.4-47.6)	147	(146.4-147.6)
			80+	10185	46	(45.2-46.8)	157	(156.2-157.8)
ALT	U/L	M	1-12	2391	9	(8.7-9.3)	32	(31.7-32.3)
			13-18	2978	8	(7.6-8.4)	38	(37.6-38.4)
			19-50	45779	10	(9.9-10.2)	55	(54.9-55.2)
			51-65	37916	11	(10.9-11.1)	51	(50.9-51.1)
			66-80	42390	9	(8.9-9.1)	43	(42.9-43.1)
			80+	21014	7	(6.9-7.1)	34	(33.9-34.1)
		F	1-12	2308	9	(8.7-9.3)	31	(30.7-31.3)
			13-18	4748	7	(6.8-7.2)	27	(26.8-27.2)
			19-50	77714	7	(6.9-7.1)	35	(34.9-35.1)
			51-65	52710	9	(8.9-9.1)	42	(41.9-42.1)
			66-80	60878	8	(7.9-8.1)	36	(35.9-36.1)
			80+	4353	6	(5.8-6.2)	29	(28.8-29.2)
AST	U/L	M	1-5	557	25	(24.2-25.8)	51	(50.2-51.8)
			6-12	921	20	(19.5-20.5)	42	(41.5-42.5)
			13-18	1262	15	(14.6-15.5)	38	(37.6-38.5)
			19+	50953	13	(12.9-13.1)	38	(37.9-38.1)

			1-5	407	26	(25.1-26.9)	51	(50.1-51.9)
		F	6-12	1035	18	(17.5-18.5)	42	(41.5-42.5)
			13-18	1980	13	(12.7-13.3)	30	(29.7-30.3)
			19+	78383	13	(12.9-13.1)	36	(35.9-36.1)
Bilirubin (total)	mg/dL (µmol/L)	M	6-12	123	0.23	(0.19-0.27)	0.84	(0.8-0.88)
			13-18	301	0.29	(0.25-0.33)	1.34	(1.30-1.38)
			19+	16323	0.32	(0.31-0.33)	1.3	(1.29-1.31)
		F	6-12	573	0.23	(0.20-0.26)	1.1	(1.07-1.13)
			19+	24215	0.28	(0.28-0.28)	1.04	(1.04-1.04)
Calcium	mg/dL (mmol/L)	M + F	1-5	318	9.2	(9.14-9.26)	10.7	(10.64-10.76)
			6-12	954	9.3	(9.27-9.33)	10.5	(10.47-10.53)
			13-18	1358	9.2	(9.18-9.22)	10.5	(10.48-10.52)
			19+	46602	8.8	(8.80-8.80)	10.3	(10.30-10.30)
Chloride	mmol/L	M + F		784	98	(97.8-98.3)	108	(107.8-108.3)
Creatinine	mg/dL (µmol/L)	M	6-12	1317	0.37	(0.37-0.37)	0.61	(0.61-0.61)
			13-18	3517	0.47	(0.46-0.48)	1.07	(1.06-1.08)
			19-50	53345	0.65	(0.65-0.65)	1.17	(1.17-1.17)
			51-65	44666	0.62	(0.62-0.62)	1.23	(1.23-1.23)
			66-80	48705	0.62	(0.62-0.62)	1.36	(1.36-1.36)
			80+	22032	0.63	(0.63-0.63)	1.53	(1.53-1.53)
		F	6-12	1364	0.37	(0.37-0.37)	0.59	(0.59-0.59)
			13-18	4804	0.45	(0.45-0.45)	0.83	(0.83-0.83)
			19-50	80957	0.47	(0.47-0.47)	0.9	(0.90-0.90)
			51-65	56832	0.47	(0.47-0.47)	0.95	(0.95-0.95)
			66-80	67350	0.46	(0.46-0.46)	1.09	(1.09-1.09)
			80+	47750	0.48	(0.48-0.48)	1.37	(1.37-1.37)
GGT	U/L	M	1-5	174	7	(6.3-7.7)	20	(19.3-20.7)
			6-12	322	9	(8.5-9.6)	23	(22.5-23.6)
			13-18	1452	8	(7.5-8.5)	36	(35.5-36.5)
			19-50	31582	9	(8.7-9.3)	79	(78.7-79.3)
			51-65	26758	12	(11.6-12.4)	95	(94.6-95.4)
			66-80	28080	11	(10.7-11.3)	84	(83.7-84.3)
			80+	13160	8	(7.6-8.4)	79	(78.6-79.4)
		F	1-5	146	8	(7.5-8.5)	17	(16.5-17.5)
			6-12	341	8	(7.5-8.5)	22	(21.5-22.5)
			13-18	2191	7	(6.7-7.3)	26	(25.7-26.3)
			19-50	48040	7	(6.9-7.1)	48	(47.9-48.1)
			51-65	35997	8	(7.8-8.2)	71	(70.8-71.2)

			66-80	40412	8	(7.8-8.2)	65	(64.8-65.2)
			80+	27174	7	(6.8-7.3)	66	(65.8-66.3)
LDH	U/L	M + F	6-12	257	359	(346.6-371.5)	643	(630.6-655.5)
			13-18	340	274	(264.2-283.8)	531	(521.2-540.8)
			19-50	2573	256	(252.5-259.5)	507	(503.5-510.5)
			51-65	1963	274	(269.9-278.1)	534	(529.9-538.1)
			66-80	2039	270	(265.6-274.4)	551	(546.6-555.4)
			80+	1539	266	(260.3-271.7)	584	(578.3-589.7)
Magnesium	mg/dL (mmol/L)	M + F		4571	1.8	(1.79-1.81)	2.4	(2.39-2.41)
Phosphate	mg/dL (mmol/L)	M	1-5	147	4.2	(4.13-4.27)	5.4	(5.33-5.47)
			6-12	405	4.2	(4.16-4.24)	5.3	(5.26-5.34)
			13-18	390	3.6	(3.54-3.66)	5.4	(5.34-5.46)
			19-50	3132	2.4	(2.37-2.43)	4.7	(4.67-4.73)
			51-65	2917	2.2	(2.17-2.23)	4.3	(4.27-4.33)
			66+	6640	2.2	(2.18-2.22)	4.2	(4.18-4.22)
		F	1-5	126	4.3	(4.23-4.37)	5.4	(5.33-5.47)
			6-12	428	4.1	(4.06-4.14)	5.3	(5.26-5.34)
			13-18	729	3.5	(3.46-3.54)	5.2	(5.16-5.24)
			19-50	5874	2.6	(2.58-2.62)	4.7	(4.68-4.72)
			51-65	7970	2.7	(2.68-2.72)	4.7	(4.68-4.72)
			66+	18548	2.6	(2.59-2.61)	4.5	(4.49-4.51)
Potassium	mmol/L	M + F		257189	3.6	(3.60-3.60)	5.1	(5.09-5.09)
Sodium	mmol/L	M + F		256775	136	(136.0-136.0)	144	(144.0-144.0)
Total protein	g/dL (g/L)	M + F		35141	6.1	(6.09-6.11)	8	(7.99-8.01)
Urea	mg/dL (mmol/L)	M	1-5	227	15	(13.6-16.4)	45	(43.6-46.4)
			6-12	755	19	(18.3-19.7)	47	(46.3-47.7)
			13-18	996	18	(17.4-18.6)	47	(46.4-47.6)
			19-50	4709	20	(19.7-20.4)	54	(53.7-54.4)
			51-65	4457	21	(20.6-21.4)	61	(60.6-61.4)
			66-80	5680	23	(22.5-23.5)	75	(74.5-75.5)
			80+	3662	27	(26.2-27.8)	93	(92.2-93.8)
		F	1-5	167	16	(14.5-17.5)	43	(41.5-44.5)
			6-12	767	17	(16.3-17.7)	44	(43.3-44.7)
			13-18	1220	16	(15.5-16.5)	42	(41.5-42.5)
			19-50	6850	16	(15.8-16.3)	46	(45.8-46.3)
			51-65	5524	20	(19.6-20.4)	58	(57.6-58.4)

66-80	6986	22	(21.6-22.4)	72	(71.6-72.4)
80+	7668	25	(24.4-25.6)	97	(96.4-97.6)
