# Supplementary material for: Indirect determination of biochemistry reference intervals using outpatient data
Source: PLoS One. 2022 May 19;17(5):e0268522. doi: 10.1371/journal.pone.0268522 (PMC9119462; doi:10.1371/journal.pone.0268522)
Supplement: S4 Table — Confidence intervals are presented for the RLE method. Results are presented in calculated and international units. (PDF) [file pone.0268522.s007.pdf]

S4 Table.

Test	Unit	Sex	Age	NUMBER n	NUMBER Low limit	NUMBER High limit	Reference limit estimator method n	Low limit	90%CI	High limit	90%CI
Albumin	g/dL (g/L)	M	18-50	4281	4.0 (40)	5.1 (51)	4352	4.0 (40)	(3.89-4.13)	5.0 (50)	(4.89-5.19)
			51-65	3660	3.8 (38)	4.9 (49)	3684	3.8 (38)	(3.70-3.96)	5.0 (50)	(4.79-5.11)
			66-80	4704	3.5 (35)	4.9 (49)	4806	3.5 (35)	(3.38-3.66)	5.1 (51)	(4.90-5.28)
			80+	4299	3.2 (32)	4.7 (47)	4721	3.2 (32)	(3.03-3.29)	4.8 (48)	(4.56-4.94)
		F	19-50	6622	3.7 (37)	4.9 (49)	6640	3.8 (38)	(3.66-3.9)	4.8 (48)	(4.69-4.99)
			51-65	5966	3.8 (38)	4.8 (48)	5960	3.8 (38)	(3.70-3.94)	4.8 (48)	(4.61-4.89)
			66-80	7865	3.6 (36)	4.7 (47)	7933	3.7 (37)	(3.56-3.8)	4.8 (48)	(4.60-4.90)
			80+	10767	3.2 (32)	4.6 (46)	11805	3.3 (33)	(3.19-3.45)	4.6 (46)	(4.47-4.81)
ALP	U/L	M	13-18	381	74	218	935	74	(68.3-78.7)	190	(178.5-200.5)
			19-50	11577	46	133	17401	47	(43.3-50.3)	133	(125.0-141.2)
			51-65	9928	45	135	14449	46	(42.9-49.9)	133	(125.0-141.2)
			66-80	9520	44	137	12880	45	(41.7-48.5)	128	(119.7-135.3)
			80+	4569	46	155	6056	46	(42.3-49.7)	147	(137.2-156.0)
		F	13-18	880	50	184	1126	52	(48.8-55.0)	108	(102.3-113.5)
			19-50	15757	39	130	19451	39	(36.1-42.3)	122	(114.0-129.4)
			51-65	14428	49	152	18368	51	(47.1-55.1)	151	(141.9-160.7)
			66-80	15285	47	147	18705	49	(45.0-52.6)	143	(133.9-151.5)
			80+	10185	46	157	12627	47	(42.8-50.4)	148	(138.6-157.4)
ALT	U/L	M	19-50	45779	10	55	54216	10	(9.3-	47	(43.4-

									11.3)		50.2)
			51-65	37916	11	51	43602	11	(10.0-12.2)	50	(46.8-54.0)
			66-80	42390	9	43	46988	10	(8.8-10.6)	39	(36.6-42.0)
			80+	21014	7	34	23185	7	(6.6-8.0)	30	(27.5-31.7)
		F	19-50	77714	7	35	82790	8	(6.9-8.3)	27	(25.2-28.8)
			51-65	52710	9	42	56864	9	(8.6-10.2)	36	(33.1-37.9)
			66-80	60878	8	36	64624	9	(8.4-10.0)	30	(28.0-31.8)
			80+	4353	6	29	46539	7	(6.0-7.2)	24	(22.8-26.0)
AST	U/L	M	1-5	557	25	51	841	24	(22.7-25.9)	56	(52.5-58.5)
			6-12	921	20	42	1401	19	(18.3-20.5)	39	(37.1-41.1)
			13-18	1262	15	38	1770	16	(14.9-16.9)	35	(32.8-36.6)
			19+	50953	13	38	65475	14	(13.0-15.0)	37	(34.8-39.2)
		F	1-5	407	26	51	655	25	(24.1-26.7)	44	(41.8-45.8)
			6-12	1035	18	42	1505	18	(16.4-18.6)	37	(35.2-39.2)
			13-18	1980	13	30	2206	14	(12.7-14.3)	28	(26.6-29.6)
			19+	78383	13	36	89705	13	(12.1-13.9)	33	(30.9-34.7)
Bilirubin (total)	mg/dL (µmol/L)	M	6-12	123	0.23 (4)	0.84 (14)	291	0.23 (4)	(0.21-0.24)	0.44 (7)	(0.41-0.46)
			13-18	301	0.29 (5)	1.34 (23)	452	0.19 (3)	(0.17-0.21)	0.88 (15)	(0.82-0.95)
			19+	16323	0.32 (6)	1.30 (22)	19086	0.31 (5)	(0.28-0.34)	1.26 (22)	(1.17-1.35)
		F	6-18	573	0.23 (4)	1.10 (19)	786	0.21 (4)	(0.19-0.23)	0.93 (16)	(0.86-1.00)
			19+	24215	0.28 (5)	1.04 (18)	26404	0.28 (5)	(0.26-0.30)	0.95 (16)	(0.89-1.01)
Calcium	mg/dL (mmol/L)	M + F	1-5	318	9.2 (2.29)	10.7 (2.67)	951	9.4 (2.33)	(9.12-9.58)	10.9 (2.71)	(10.61-11.13)
			6-12	954	9.3 (2.32)	10.5 (2.63)	1686	9.4 (2.35)	(9.22-9.58)	10.4 (2.59)	(10.18-10.58)
			13-18	1358	9.2 (2.29)	10.5 (2.61)	1629	9.2 (2.31)	(9.03-9.45)	10.5 (2.62)	(10.27-10.73)
			19+	46602	8.8 (2.20)	10.3 (2.58)	60579	8.9 (2.21)	(8.65-9.09)	10.3 (2.57)	(10.05-

										10.53)
Chloride	mmol/L	M + F		784	98	108	883	99	(97.2-100.8)	108	(106.1-109.9)
Creatinine	mg/dL (µmol/L)	M	19-50	53345	0.65 (57)	1.17 (103)	53215	0,65 (57)	(0.61-0.68)	1.12 (99)	(1.07-1.17)
			51-65	44666	0.62 (54)	1.23 (109)	44921	0.63 (56)	(0.60-0.67)	1.17 (103)	(1.11-1.22)
			66-80	48705	0.62 (55)	1.36 (121)	50515	0.64 (57)	(0.60-0.68)	1.28 (114)	(1.22-1.35)
			80+	22032	0.63 (56)	1.53 (135)	25461	0.63 (55)	(0.58-0.67)	1.53 (136)	(1.45-1.62)
		F	19-50	80957	0.47 (41)	0.90 (79)	81069	0.46 (41)	(0.44-0.49)	0.86 (76)	(0.81-0.90)
			51-65	56832	0.47 (41)	0.95 (84)	56770	0.48 (42)	(0.45-0.50)	0.89 (79)	(0.85-0.93)
			66-80	67350	0.46 (41)	1.09 (96)	67772	0.48 (43)	(0.45-0.51)	0.99 (88)	(0.94-1.04)
			80+	47750	0.48 (42)	1.37 (121)	50542	0.49 (43)	(0.45-0.52)	1.21 (107)	(1.14-1.27)
GGT	U/L	M	19-50	31582	9	79	38692	9	(8.4-10.4)	52	(48.2-56.2)
			51-65	26758	12	95	31762	12	(10.5-13.3)	72	(66.5-77.9)
			66-80	28080	11	84	31909	12	(10.6-13.0)	53	(48.8-56.4)
			80+	13160	8	79	14901	9	(8.3-10.3)	48	(44.4-51.6)
		F	19-50	48040	7	48	51958	8	(7.3-8.7)	27	(24.9-28.3)
			51-65	35997	8	71	39690	9	(7.7-9.5)	42	(39.1-45.3)
			66-80	40412	8	65	43540	10	(8.9-10.5)	34	(32.0-36.6)
			80+	27174	7	66	29585	9	(8-9.6.0)	35	(32.6-37.4)
LDH	U/L	M + F	6-12	257	359	643	273	332	(313.2-350.8)	645	(613.4-677.4)
			13-18	340	274	531	340	288	(275.5-299.9)	432	(414.6-448.4)
			19-50	2573	256	507	2607	260	(246.1-273.5)	467	(445.0-488.6)
			51-65	1963	274	534	1983	282	(266.9-296.3)	503	(479.6-526.4)
			66-80	2039	270	551	2077	273	(257.7-287.7)	512	(487.1-536.5)

			80+	1539	266	584	1598	270	(253.6 - 286.0)	562	(532.9 - 591.3)
Magnesium	mg/dL (mmol/L)	M + F		4571	1.8 (0.72)	2.4 (1.00)	4915	1.8 (0.75)	(1.77-1.89)	2.4 (0.99)	(2.32-2.47)
Phosphate	mg/dL (mmol/L)	M	1-5	147	4.2 (1.34)	5.4 (1.74)	476	4.7 (1.49)	(4.48-4.82)	6.3 (2.00)	(6.04-6.46)
			6-12	405	4.2 (1.33)	5.3 (1.7)	763	4.2 (1.34)	(4.02-4.38)	6.3 (2.01)	(6.03-6.53)
			13-18	390	3.6 (1.16)	5.4 (1.72)	605	3.9 (1.24)	(3.70-4.06)	6.2 (2.00)	(5.98-6.50)
			19-50	3132	2.4 (0.77)	4.7 (1.51)	3172	2.4 (0.77)	(2.29-2.55)	4.6 (1.48)	(4.39-4.85)
			51-65	2917	2.2 (0.72)	4.3 (1.38)	2953	2.5 (0.79)	(2.33-2.59)	4.4 (1.40)	(4.19-4.59)
			66+	6640	2.2 (0.71)	4.2 (1.34)	6885	2.5 (0.78)	(2.33-2.57)	4.2 (1.35)	(4.04-4.42)
		F	1-5	126	4.3 (1.39)	5.4 (1.71)	430	4.5 (1.45)	(4.34-4.70)	6.5 (2.07)	(6.23-6.71)
			6-12	428	4.1 (1.32)	5.3 (1.71)	807	4.2 (1.35)	(4.05-4.39)	6.2 (1.97)	(5.92-6.38)
			13-18	729	3.5 (1.11)	5.2 (1.67)	791	3.6 (1.16)	(3.48-3.78)	5.4 (1.72)	(5.17-5.59)
			19-50	5874	2.6 (0.84)	4.7 (1.51)	5934	2.7 (0.86)	(2.55-2.83)	4.8 (1.53)	(4.55-4.99)
			51-65	7970	2.7 (0.88)	4.7 (1.5)	8041	3.0 (0.96)	(2.85-3.13)	4.8 (1.52)	(4.55-4.95)
			66+	18548	2.6 (0.84)	4.5 (1.43)	19113	2.8 (0.90)	(2.68-2.94)	4.5 (1.44)	(4.32-4.70)
Potassium	mmol/L	M + F		257189	3.6	5.1	269303	3.7	(3.54-3.82)	5.1	(4.89-5.24)
Sodium	mmol/L	M + F		256775	136	144	268873	136	(134.6 - 138.4)	144	(142.1 - 146.0)
Total protein	g/dL (g/L)	M + F		35141	6.1 (61)	8.0 (80)	36883	6.2 (62)	(6.04-6.45)	8.0 (80)	(7.75-8.24)
Urea	mg/dL (mmol/L)	M	1-5	227	15 (2.5)	45 (7.5)	618	13 (2.1)	(11.5-13.5)	41 (6.8)	(38.4-43.6)
			6-12	755	19 (3.1)	47 (7.7)	1094	18 (3.0)	(16.8-19.4)	47 (7.8)	(44.2-49.6)
			13-18	996	18 (3.0)	47 (7.8)	1001	17 (2.7)	(15.3-17.7)	45 (7.5)	(42.2-47.6)
			19-50	4709	20 (3.3)	54 (9.0)	4795	20 (3.3)	(18.3-21.1)	51 (8.6)	(48.4-54.4)
			51-65	4457	21 (3.5)	61 (10.2)	4862	20 (3.3)	(18.4-21.4)	56 (9.3)	(52.6-59.4)
			66-80	5680	23 (3.9)	75 (12.6)	7157	25 (4.1)	(22.6-26.4)	75 (12.4)	(70.0-79.4)

			80+	3662	27 (4.5)	93 (15.5)	6347	28 (4.6)	(25.1-30.7)	131 (21.8)	(121.4 - 140.4)
		F	1-5	167	16 (2.6)	43 (7.2)	499	14 (2.3)	(12.7-14.7)	37 (6.1)	(34.7-39.1)
			6-12	767	17 (2.8)	44 (7.4)	1168	16 (2.7)	(15.2-17.6)	43 (7.2)	(40.7-45.7)
			13-18	1220	16 (2.7)	42 (7.0)	1240	15 (2.5)	(13.7-15.9)	38 (6.4)	(36.2-40.6)
			19-50	6850	16 (2.7)	46 (7.7)	7001	16 (2.6)	(14.7-17.1)	43 (7.1)	(40.1-45.1)
			51-65	5524	20 (3.4)	58 (9.7)	5630	20 (3.4)	(18.8-21.8)	56 (9.2)	(52.2-58.8)
			66-80	6986	22 (3.7)	72 (12.0)	7447	24 (3.9)	(22.0-25.4)	65 (10.8)	(60.7-68.5)
			80+	7668	25 (4.1)	97 (16.2)	9801	24 (4.0)	(21.9-26.3)	98 (16.3)	(91.0-104.6)
CK	U/L	M	13-18	70	55	384	76	38	(33.5-41.9)	210	(193.9 - 226.1)
			19-50	772	50	380	1571	47	(40.6-52.8)	364	(333.4 - 394.0)
			51-65	1073	42	331	2057	43	(37.5-48.1)	296	(272.1 - 320.1)
			66-80	1057	33	280	1072	26	(23.4-28.2)	107	(99.1-114.1)
			80+	383	27	214	394	30	(26.6-33.0)	159	(146.6 - 170.6)
		F	13-18	69	37	225	67	47	(43.5-49.9)	116	(109.5 - 122.9)
			19-50	808	32	209	1579	36	(32.2-39)	155	(144.0 - 166.2)
			51-65	1162	34	238	2311	38	(34.0-41.0)	150	(139.9 - 160.7)
			66-80	1329	30	213	1333	28	(25.8-30.8)	102	(95.3-108.9)
			80+	726	23	199	745	17	(14.4-19.4)	172	(156.9 - 187.5)
Uric Acid	mg/dL (mmol/L)	M	6-12	117	2,1 (0,12)	5,6 (0,34)	115	2.2 (0.13)	(2.00-2.30)	5.3 (0.31)	(4.95-5.55)
			13-18	660	3,3 (0,20)	7,9 (0,47)	634	3.5 (0.21)	(3.28-3.72)	7.5 (0.45)	(7.11-7.91)
			19-50	22950	3,4 (0,20)	8,4 (0,50)	23022	3.9 (0.23)	(3.61-4.11)	8.7 (0.52)	(8.24-9.18)
			51-65	30690	3,5 (0,21)	8,7 (0,52)	30909	3.8 (0.22)	(3.52-4.02)	9.0 (0.54)	(8.50-9.52)

	66-80	38168	3,4 (0,21)	8,8 (0,53)	38597	3.8 (0.23)	(3.58-4.10)	9.5 (0.56)	(8.95-10.03)
	80+	18803	3,3 (0,20)	9,0 (0,54)	19382	3.6 (0.21)	(3.29-3.81)	9.4 (0.56)	(8.86-9.98)
F	6-12	110	1,9 (0,11)	5,8 (0,35)	110	1.9 (0.11)	(1.76-2.04)	5.4 (0.32)	(5.05-5.71)
	13-18	790	2,4 (0,15)	6,1 (0,36)	771	2.5 (0.15)	(2.32-2.64)	5.7 (0.34)	(5.41-6.05)
	19-50	28283	2,2 (0,13)	6,4 (0,38)	28138	2.6 (0.15)	(2.39-2.73)	6.1 (0.37)	(5.79-6.49)
	51-65	33556	2,4 (0,14)	7,3 (0,44)	33398	2.9 (0.17)	(2.68-3.10)	7.5 (0.45)	(7.10-7.98)
	66-80	47549	2,5 (0,15)	7,8 (0,47)	47587	3.1 (0.18	(2.86-3.32)	8.4 (0.50)	(7.92-8.92)
	80+	36558	2,5 (0,15)	8,6 (0,51)	37517	3.1 (0.18)	(2.80-3.330)	9.8 (0.58)	(9.20-10.46)
